# Emotionally Structured Interaction Networks and Consumer Perception of New Energy Vehicle Technology: A Behavioral Network Analysis of Online Brand Communities

**DOI:** 10.3390/bs16010112

**Published:** 2026-01-14

**Authors:** Jia Xu, Chang Liu, Liangdong Lu

**Affiliations:** Business School, Hohai University, Nanjing 211100, China; jiaxu@hhu.edu.cn (J.X.); changliu0912@hhu.edu.cn (C.L.)

**Keywords:** consumer perception, emotional homophily, behavioral network analysis, new energy vehicles (NEVs), online brand communities

## Abstract

This study investigates how emotionally structured online interaction networks shape consumer perception of new energy vehicle (NEV) technology. Drawing on discussion forum data from two leading NEV brands, Brand_T and Brand_B, we focus on how users respond to brand technological narratives and how these responses translate into distinct patterns of peer-to-peer interaction. Using a behavioral network analysis framework, we integrate sentiment analysis, topic modeling, and Exponential Random Graph Modeling (ERGM) to uncover the psychological and structural mechanisms underlying consumer engagement. Three main findings emerge. First, users display brand-specific emotional-cognitive profiles: Brand_T communities show broader technological engagement but more heterogeneous emotional responses, whereas Brand_B communities exhibit more emotionally aligned discussions. Second, emotional homophily is a robust driver of interaction ties, particularly in Brand_B forums, where positive sentiment clusters into dense and supportive discussion subnetworks. Third, perceived technological benefits, rather than risk sensitivity, are consistently associated with higher interaction intensity, underscoring the motivational salience of anticipated gains over cautionary concerns in shaping engagement behavior. The study contributes to behavioral science and transportation behavior research by linking consumer sentiment, cognition, and social interaction dynamics in digital environments, offering an integrated theoretical account that bridges the Elaboration Likelihood Model, social identity processes, and behavioral network formation. This advances the understanding of technology perception from static individual evaluations to dynamic, group-structured outcomes. It highlights how emotionally patterned interaction networks can reinforce or recalibrate technology-related perceptions, offering practical implications for NEV manufacturers and policymakers seeking to design psychologically informed communication strategies that support sustainable technology adoption.

## 1. Introduction

As global demand for sustainable energy solutions intensifies, the new energy vehicle (NEV) sector has emerged as a key driver of technological innovation and environmental progress (Luthra et al., 2015). Yet, the widespread adoption of NEVs depends not only on technological advancements but also on how these innovations are perceived by consumers (Crisafulli et al., 2025). Effective brand communication plays a vital role in shaping such perceptions by building trust and reducing uncertainty associated with novel technologies (Siegrist, 2000; Boukis, 2020). In particular, consumer perceptions are often influenced less by objective performance metrics than by affective evaluations, peer recommendations, and social media narratives (C. Chen et al., 2024).

In the current media ecosystem, social media platforms have replaced traditional communication channels as the dominant space for consumer-brand engagement (Kapoor et al., 2018; Song & Kim, 2025). Within these interactive environments, users are not passive recipients of information but active contributors to emotionally charged discussions. These interactions often give rise to emotionally aligned clusters, where individuals primarily encounter opinions and sentiments that reinforce their existing views (Garrett, 2009; Cinelli et al., 2021). While such emotional convergence may enhance message persuasiveness and user engagement, it can also narrow exposure to diverse perspectives, influencing how consumers evaluate technologies (Wang & Song, 2020).

Although the role of social media in shaping public attitudes toward innovation has attracted scholarly attention, relatively little research has examined how emotion-driven interactions within brand-specific communities shape consumer perceptions of NEV technologies. Most existing studies focus on risk communication, information diffusion, or individual acceptance models, without considering the collective emotional and cognitive structures that emerge in online discussions (Garg et al., 2011; Slade et al., 2015). Furthermore, recent studies in transportation behavior suggest that group-level dynamics such as emotional homophily and cognitive alignment can significantly influence mobility-related attitudes and behaviors, particularly in digital contexts (Fu, 2024; Bretter & Pangbourne, 2025).

To address this gap, the present study explores how emotional tendencies, cognitive traits, and perceived benefits influence consumer interaction patterns in NEV-related online forums. We focus on two major brands, Brand_T and Brand_B, whose distinct market positioning and consumer engagement strategies provide a useful contrast for examining brand-specific emotional and behavioral dynamics. Brand_T’s emphasis on innovation and elite technological appeal may attract more critical or aspirational users, while Brand_B’s positioning as an accessible and eco-conscious brand may foster more affectively homogeneous and community-oriented engagement.

We adopt a behavioral network analysis approach that integrates sentiment analysis, topic modeling, and Exponential Random Graph Modeling (ERGM). This methodology enables us to uncover how user attributes, such as emotional valence, cognitive orientation, and benefit perception, shape peer interactions and influence the emotional structure of online communities. In doing so, the study contributes to a deeper understanding of the affective and relational mechanisms underlying transportation technology acceptance. Our findings offer practical implications for NEV manufacturers and policymakers in designing emotionally attuned and behaviorally informed communication strategies that can promote broader public acceptance of sustainable mobility technologies.

Based on the above objectives, we formulate the following research questions:

RQ1: How do emotional and cognitive traits influence consumer interactions within online NEV communities?

RQ2: How do different brand technological narratives trigger divergent emotional patterns and discussion behaviors among consumers?

RQ3: What psychological attributes (e.g., emotional valence, perceived benefit) are associated with increased likelihood of user-to-user interaction in NEV forums?

## 2. Literature Review and Theoretical Foundation

### 2.1. Brand Technological Communication and Consumer Perception

Brand technological communication plays a pivotal role in shaping how consumers understand and emotionally evaluate innovative vehicle technologies (Costello & Kim, 2025). In high-involvement product categories like new energy vehicles (NEVs), effective communication not only enhances awareness of technical features but also builds trust and reduces perceived uncertainty (Chiesa & Frattini, 2011). This process is especially critical given that NEVs often involve unfamiliar functions, such as battery management systems, autonomous driving assistance, and connected interfaces.

The emotional dimension of brand technological communication is integral to its narrative. Consumers are more likely to adopt complex technologies when information is transparent, authentic, and emotionally resonant (Keiningham et al., 2020; Mantello et al., 2023). This resonance stems from aligning brand messages with consumers’ psychological needs, values, and aspirations, particularly concerning safety, ecological responsibility, and innovation identity (Crisafulli et al., 2025). For instance, brands that successfully integrate sustainability narratives into their technological framing are more likely to build trust among environmentally conscious consumers (Schmidt et al., 2017). In contrast, strategies focusing solely on functional benefits may prove inadequate if they neglect these emotional and psychological drivers of decision-making (Schmitt et al., 2015). NEV consumers are not just evaluating mileage or charge time; they are making judgments about safety, environmental commitment, and technological leadership. Therefore, brand communication serves both an informational and affective function, shaping the mental models that underlie consumer perception and behavior (Dai et al., 2025).

The digital landscape, particularly social media, has fundamentally transformed communication process by allowing for two-way communication and the co-construction of brand meaning between companies and users, often in emotionally charged contexts (Brodie et al., 2013). Brands leverage these platforms for interactive engagement, utilizing testimonials and data visualizations to reinforce the perceived credibility of new technologies (Hennig-Thurau et al., 2004). Beyond brand-led communication, user-generated content (UCG), such as reviews, reactions, and peer recommendations, actively contributes to and reframes the technological narrative within their social networks, adding emotional tone and normative cues (Ibrahim et al., 2025).

Thus, affective trust and emotional identification fostered through both compelling brand technological communication and peer-driven UGC significantly shape the perceived value of NEV technology, often outweighing purely rational assessments (Jang et al., 2021; Tran et al., 2024). Understanding this dynamic is essential for crafting strategies that foster emotionally congruent communities and promote the acceptance of NEV technologies through trust-based engagement.

### 2.2. Social Identity and Emotional Interaction Patterns in Online Communities

Emotional dynamics in online communities are closely tied to social identity mechanisms. Social identity theory posits that individuals derive their sense of self and belonging from their membership in social groups, reinforcing in-group loyalty and out-group distancing (Tajfel & Turner, 2004; Hogg & Rinella, 2018). In the context of NEVs, Consumers often gravitate toward and participate in online communities composed of emotionally similar peers—a phenomenon known as emotional homophily—based on shared attitudes, beliefs, or brand preferences (Hobolt et al., 2024). This self-categorization into emotionally congruent groups forms the foundational social identity mechanism driving subsequent affective and behavioral patterns (Bulboacă et al., 2025).

Over time, these emotionally congruent groups develop sentiment clustering, wherein positive or negative feelings toward a brand or technology are continuously reinforced (Scheufele & Krause, 2019). This process is driven by cognitive and social psychological dynamics, such as the need to reduce cognitive dissonance and enhance interpersonal trust through validation from similar others (Isenberg, 1986; Matz & Wood, 2005; Páez et al., 2015). In online NEV discussions, this implies that users may selectively engage with content, and with other users, that validates their existing feelings about the brand. This emotionally selective exposure can strengthen brand loyalty but also distort perceptions by limiting critical perspectives, as they may repeatedly highlight its advantages, share positive testimonials, and marginalize negative feedback (Lee et al., 2009; K. J. Chen et al., 2015).

This identity-based emotional bonding is further intensified by the technological architecture of social platforms. Emotional valence in social platforms often interacts with algorithmic filtering mechanisms, reinforcing homogeneous feedback loops (K. J. Chen et al., 2015; Just & Latzer, 2017). While these loops can generate rapid technology adoption through social proof, they may also amplify unrealistic optimism or overemphasize rare failures (Kitchens et al., 2020). These dynamics mirror findings from studies on digital misinformation and affective polarization, where high-valence content tends to receive more algorithmic amplification, thus distorting collective perception.

Understanding these affective structures is essential for evaluating how NEV technologies are collectively framed and accepted. It also calls for strategic interventions by brands and policymakers to encourage more emotionally diverse discussions, mitigate affective silos, and build trust across a broader spectrum of consumer sentiment.

### 2.3. The Elaboration Likelihood Model and Behavioral Network Perspective on Technology Acceptance

The Elaboration Likelihood Model (ELM) provides a robust framework for examining how diverse user attributes influence information processing and social connection formation in online technology discussions. ELM distinguishes between two routes to persuasion: the central route, characterized by effortful cognitive elaboration of argument quality (e.g., perceived benefits and risks), and the peripheral route, reliant on heuristic cues such as emotion (Petty et al., 1981; Bhattacherjee & Sanford, 2006; Shao et al., 2023; Zhang & Hu, 2024). In the context of NEV online communities, user interactions are influenced by a constellation of attributes that map onto these dual pathways, collectively shaping the dynamic process from information reception and sharing, to the establishment of homophilous ties, and ultimately to technology acceptance.

To empirically investigate how ELM-relevant attributes, which include emotion (a peripheral cue), cognitive language use (a central processing indicator), and perceptions of benefits and risks (argument quality), govern relational dynamics, we adopt a behavioral network perspective complemented by Exponential Random Graph Modeling (ERGM). This approach focuses on the structural patterns of interaction, modeling and analyzing how specific attributes influence the likelihood of forming connections with others, thus sheds light on how online discussions around NEVs become emotionally polarized or harmonized over time, beyond what traditional sentiment analysis can capture (Liu & Zeng, 2024; Xu et al., 2025).

This integrated perspective aligns with the finding that emotional alignment can be a more decisive force than factual content in forging digital social bonds, often operating as a powerful peripheral route (Luo et al., 2023). Simultaneously, the spread and reinforcement of affective states through networks can amplify shared sentiments and drive behavioral convergence, making collective perception a function of socially reinforced emotional consensus rather than solely individual analysis (Yin et al., 2024; Im et al., 2025). Consequently, a consumer’s stance toward NEV technology perception is relationally constructed through networked exchanges, where attributes tied to both peripheral and central processing interact, echoing the affective contagion and cognitive alignment within brand communities (Tecău et al., 2025).

By applying this ELM-informed, attribute-focused behavioral network framework, we advance a nuanced understanding of technology acceptance. It posits that the formation of opinion clusters and information diffusion pathways in NEV communities can be traced to the differential roles of key user attributes within the dual-processing landscape. This approach not only provides a richer theoretical synthesis of ELM and network theory but also offers actionable insights for designing communication that effectively engages both the cognitive and emotional drivers of consumer perception and connection.

In summary, existing research has established that brand technological communication is crucial in shaping consumer perceptions of technology, with the affective dimension and user participation in brand narratives being particularly key in the current digital environment. These narratives and interactions unfold within online communities governed by social identity processes, where emotional homophily and algorithmically reinforced feedback loops lead to sentiment clustering and selective exposure, collectively constructing technology perception. Furthermore, the Elaboration Likelihood Model combined with a behavioral network perspective provides a solid analytical foundation for examining how key user attributes influence both technology perception and the formation of social ties.

Nevertheless, few studies have examined how distinct brand narrative themes and the resulting emotional clusters shape the structure of peer interactions, specifically within the context of NEV online community platforms. To address this gap, the present study integrates the dual-process theory of the Elaboration Likelihood Model with social identity and behavioral network perspectives. It employs advanced methods such as Exponential Random Graph Modeling to systematically test how specific attributes influence the directional patterns of connections, including receiving and sending information, as well as the formation of homophilous ties. This approach facilitates a more nuanced understanding of technology acceptance and offers actionable insights for designing communication strategies that effectively engage the cognitive and affective drivers of consumer perception and behavior.

## 3. Methods

### 3.1. Data Collection and Sample Description

To investigate the behavioral mechanisms underlying consumer perception in the NEV market, we collected large-scale user-generated content from Autohome, one of China’s largest automotive discussion forums. Our dataset comprises 368,048 posts and replies related to 466 NEV models across 132 brands, spanning forum discussions between 2021 and 2023. For in-depth comparative analysis, we selected Brand_T and Brand_B, two leading NEV brands in China with distinctive market positions and consumer bases.

Brand_T, with its core identity as a pioneering innovator and purveyor of elite technology, may attract users who exhibit higher cognitive engagement yet more heterogeneous affective evaluations. This could foster more contentious and critically diverse discussions within its brand community. In contrast, the established positioning of Brand_B around mass-market sustainability and ecological responsibility is more likely to attract users who share common value propositions, thereby facilitating the formation of a tightly knit and supportive interaction network characterized by stronger emotional homophily through social identity processes. Consequently, comparing these two brand communities allows for an empirical examination of how distinct brand technological narratives, by shaping different group identities, systematically influence internal affective structures, patterns of homophilous interaction, and ultimately the pathways through which technology perceptions are formed.

We focused on forums where discussions predominantly revolved around technological features and driving experiences. Each forum thread includes a primary post initiated by an original poster (OP) and a chain of replies. This structure enabled the construction of directed social interaction networks, where a reply from user j to user i is treated as a directed edge from j to i, indicating an act of attention or engagement.

Basic statistics of the selected sub-forums are shown in Table 1. Brand_B and Brand_T forums demonstrated substantial differences in user volume, post frequency, and interaction depth, offering a valuable contrast for cross-brand analysis of emotional dynamics and network structure.

### 3.2. Emotional and Cognitive Feature Extraction

To analyze the psychological attributes embedded in user interactions, we employed text mining and psycholinguistic analysis techniques. All user-generated content was processed using Python 3.12.3 and the LIWC-2015 lexicon, which classifies language features into psychologically meaningful categories such as emotion, cognition, health, risk, and informal speech.

Each user’s emotional tendency was quantified by averaging the positivity score of their messages, scaled between 0 and 1. Users were thus assigned a continuous emotional valence score reflecting their overall affective orientation toward NEVs. Similarly, attributes such as perceived benefit, cognitive processing, and risk-related concern were also extracted. To enhance transparency in the operationalization of these psychological variables, Table 2 provides illustrative vocabulary examples corresponding to each attribute category, demonstrating how these theoretical constructs are translated into measurable linguistic features.

In parallel, we applied Latent Dirichlet Allocation (LDA) to extract discussion themes for each brand, helping us understand the dominant technological topics that framed user sentiment.

To assess emotional alignment between users, we further examined the joint distribution of emotional tendencies between each user and their neighbors in the interaction network. Each user’s emotional score was computed as the mean sentiment of their posts, ranging from 0 to 1. By plotting these joint distributions, we could visualize the extent to which emotionally similar users tend to connect, an indication of emotional homophily. A concentration of data points along the diagonal reflects high emotional alignment, while a dispersed distribution suggests affective diversity. This analysis offers a system-level view of how sentiment structures interpersonal connections in brand-specific online communities.

### 3.3. Behavioral Network Construction and ERGM Modeling

We constructed user interaction networks for each brand based on reply relationships in the forum threads. In this directed graph, each node represents a user, and each edge indicates a communication act. These networks capture both the flow of information and the embedded emotional signals exchanged between users.

To understand the formation mechanism of user connections, we employed Exponential Random Graph Models (ERGMs). ERGMs are well-suited for behavioral network analysis as they allow the modeling of how user attributes (e.g., emotion, benefit perception) and structural tendencies (e.g., reciprocity, transitivity) influence the likelihood of tie formation (Robins et al., 2007; Lewis et al., 2012).

We did not use ERGM to test for the existence of echo chambers. Instead, our goal was to determine which psychological traits increase the probability of interaction between users, thereby contributing to emotional alignment or divergence. Specifically, we examined whether users with similar emotional or cognitive profiles were more likely to connect, thereby capturing homophily effects in online interactions. In addition, we investigated whether certain psychological traits, such as emotional valence, perceived benefits, or cognitive tendencies, increased a user’s likelihood of initiating communication (sender effects) or being the target of replies (receiver effects), thus revealing asymmetries in interaction behavior based on individual characteristics.

These ERGM terms help reveal the behavioral pathways through which emotional congruence, technological optimism, or risk sensitivity contribute to network structure. Models were estimated using the Statnet package in R, and model fit was assessed via AIC/BIC and goodness-of-fit diagnostics.

## 4. Results

### 4.1. Consumer Engagement with Brand Technological Narratives

To assess the effectiveness of brand technological communication, we compared the proportion of brand-promoted technologies that were echoed in consumer-generated discussions. We compiled official website texts from 10 NEV brands, extracting the technological elements emphasized in their promotional materials. These were matched with user discussions on forums, enabling a calculation of “coverage rate”, i.e., how much of a brand’s technological narrative is reflected in consumer discourse.

The findings show that Brand_T exhibited the highest coverage rate (0.551), indicating a strong alignment between its promotional communication and consumer discussion. Brand_B showed a lower coverage rate (0.385), suggesting a partial gap between official messaging and user-level perception. This contrast may reflect differences in communication clarity, emotional resonance, or the demographic and psychological profiles of each brand’s consumer base.

This finding implies that consumer attention and engagement with brand messaging are not only driven by the availability of information but also by the emotional and cognitive accessibility of that information. Brand_T’s branding appears to trigger more widespread and emotionally charged discussion among users, potentially reinforcing social validation effects that promote brand-specific loyalty and confidence.

### 4.2. Differences in Consumer Attention and Cognitive Framing

To further explore how consumers interpret technological narratives, we conducted topic modeling using Latent Dirichlet Allocation (LDA) on forum discussions related to Brand_T and Brand_B. For Brand_B, six distinct themes emerged (e.g., driving experience, model performance, innovation, maintenance), while Brand_T generated thirteen topics with broader and more detailed coverage, including international market strategies, intelligent systems, and battery performance (see Table 3 and Table 4).

This divergence suggests that Brand_T users engage with NEV technology at a more granular and diversified cognitive level, potentially reflecting higher levels of technological familiarity, curiosity, or identity alignment with innovation. Brand_B discussions, while rich in content, tended to concentrate on core functionality and product value, indicating a more utility-oriented or pragmatic approach to technological assessment.

These findings support the idea that consumer perception is shaped not only by the technological message itself but also by how the user community cognitively frames and emotionally filters that information. In particular, Brand_T’s diverse topic range may contribute to a broader spectrum of user emotions and interactions, setting the stage for the behavioral network patterns explored in subsequent sections.

### 4.3. Emotional Patterns and Interaction Structures Across Brand Communities

Building upon these cognitive themes, we now examine the emotional dynamics underlying consumer engagement in NEV forums. Using a lexicon-based sentiment scoring method, we assessed the emotional valence of user-generated posts across brand communities. The results show that Brand_B discussions were characterized by predominantly positive sentiment, whereas Brand_T forums exhibited a more heterogeneous emotional landscape, with a higher proportion of negative or ambivalent content. These differences suggest that emotional tone may play a distinct role in shaping the structure and intensity of user interactions across brands.

**Figure 1:**
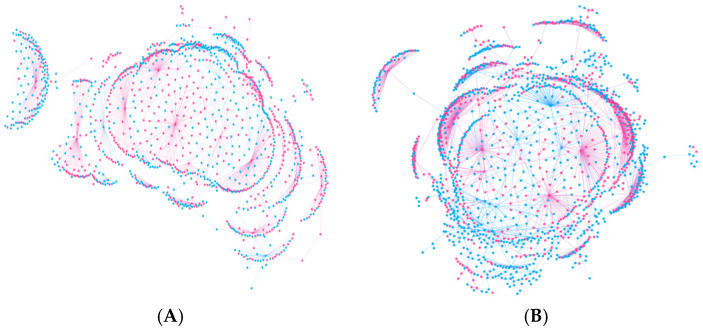
Emotion-Oriented Interaction Networks in Brand_B and Brand_T Forums ((**A**): Brand_B, (**B**): Brand_T), with Negative Emotions in Blue and Positive Emotions in Red.

We visualized these differences through user interaction networks (Figure 1). In Brand_B’s network, users with high emotional positivity (red nodes) were central and heavily interconnected, suggesting that positive emotions facilitated interaction and network expansion. In contrast, Brand_T’s user network was more fragmented, with nodes showing negative emotions (blue) appearing more frequently and forming isolated or loosely connected subgroups.

**Figure 2:**
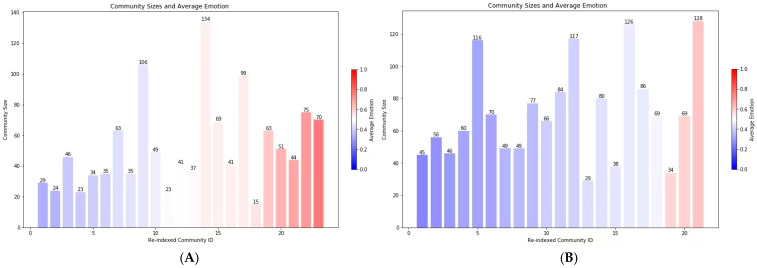
Community Clustering by Emotional Profiles in Brand_B and Brand_T Forums ((**A**): Brand_B, (**B**): Brand_T).

Further community detection analysis (Figure 2) revealed that Brand_B’s forum sustained multiple emotion-aligned clusters, where larger communities were formed around shared positive affect. These clusters displayed high modularity, indicating well-separated emotional subgroups with dense intra-group ties and sparse inter-group connections. In particular, several core modules centered around highly active users who repeatedly posted emotionally positive content, reinforcing collective sentiment and generating strong local echo effects. The network structure showed high clustering coefficients and a visibly modular topology, suggesting stable emotional cohesion and efficient internal communication.

In contrast, Brand_T’s community structure showed smaller and more fragmented clusters with lower modularity. Emotional alignment was weaker, and interactions were more individually dispersed, reflecting a loosely connected network where sentiments were less synchronized. The presence of multiple weakly connected nodes and absence of dominant emotional hubs suggest that discussions in Brand_T’s forum were more heterogeneous, possibly driven by divergent expectations or contested evaluations of brand technology.

**Figure 3:**
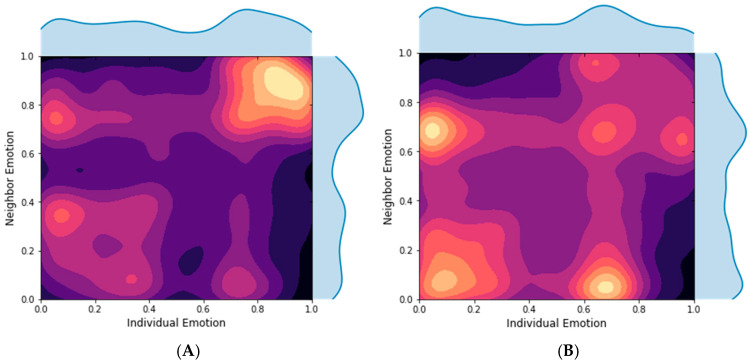
Joint Emotional Distributions Between Users and Their Neighbors ((**A**): Brand_B, (**B**): Brand_T). Colors represent the density of users: The lighter the color, the larger the number of users.

To assess emotional influence at the dyadic level, we computed joint distributions of each user’s emotional score with those of their neighbors (Figure 3). In Brand_B’s forum, the distribution was concentrated along the diagonal, indicating a high degree of emotional homophily—users with similar affective orientations were more likely to interact. This homogeneity supports the formation of positive feedback loops, where repeated reinforcement of shared emotion strengthens group coherence and amplifies collective endorsement of brand narratives.

Conversely, Brand_T’s joint sentiment distribution was more dispersed, with no dominant pattern of alignment. This reflects a lower network assortativity with respect to emotional valence, indicating that users interacted across a broader range of sentiment expressions. Such heterogeneity may contribute to ambivalent or polarized consumer discourse, which, while more diverse, may also dilute the clarity of technological messaging and reduce perceived consensus within the community.

These results suggest that emotionally congruent communities are more likely to form sustained, reciprocal interactions, particularly in environments where brand identity evokes strong affective resonance. The structural properties of these networks, such as modularity, density, and assortativity, offer valuable insights into how emotional alignment translates into behavioral cohesion. From a communication strategy perspective, cultivating emotionally resonant sub-communities may enhance message diffusion and increase brand trust within consumer networks.

### 4.4. Behavioral Mechanisms of Interaction: ERGM Findings

To explore what factors drive user-to-user interactions within NEV communities, we applied ERGMs incorporating user-level psychological attributes extracted from text (Table 5). Our aim was not to model general network structure per se, but to uncover how emotional, social, and cognitive traits shape the likelihood of connection between users.

In the Brand_B forum, users with similar emotional tendencies were significantly more likely to interact (homophily effect = 2.30, *p* < 0.01), indicating that positive emotions serve as a basis for community bonding. Moreover, users with more positive emotions were more likely both to initiate communication (sender effect = 0.45) and to receive responses (receiver effect = 0.36). Brand_T, in contrast, showed weaker or negative homophily, suggesting that emotional similarity played a lesser role in facilitating connections.

Brand_T users with strong social activity profiles were more likely to form connections (homophily = 0.65), initiate replies (sender = 0.02), and participate in information exchange. In Brand_B’s case, social homophily was negative, implying a more democratized interaction landscape where users with varying levels of connectivity still engaged. This indicates that Brand_T’s community structure favors socially active individuals, whereas Brand_B fosters broader inclusion.

Perceived benefit emerged as a consistent driver of interaction. Users with higher benefit orientation were more likely to connect in both communities, especially in Brand_B (homophily = 1.72). Conversely, users with high risk perception tended to avoid forming links with similarly concerned peers, possibly reflecting an avoidance tendency or communication breakdown around negative expectations.

Cognitive attributes, indicators of analytical or reflective language, were positively associated with both sending and receiving information. However, users with similar cognitive levels were less likely to connect (Brand_B: −0.93), suggesting that information seekers and information providers occupy different positions, reflecting a division between “knowledge contributors” and “observers”.

These ERGM findings confirm that emotional congruence and benefit perception are key behavioral mechanisms in forming NEV-related discussions. Brand_T’s interaction patterns are more selective, driven by social centrality and cognitive signaling, while Brand_B’s networks reflect a more emotionally cohesive and accessible user community. This difference aligns with their branding styles, Brand_T as a cutting-edge tech innovator and Brand_B as a mass-market sustainable mobility provider.

## 5. Conclusions and Discussion

### 5.1. Divergent Brand Narratives and Distinct Community Engagement Patterns

This study reveals distinct patterns in brand technological narratives and their corresponding community discussions. Comparative analysis indicates that the “innovative pioneer” narrative adopted by Brand_T fosters an in-group identity that prioritizes technological exploration and critical thinking, attracting users with high cognitive needs. This orientation encourages community discussions to proceed primarily through the central route of the Elaboration Likelihood Model, manifesting as more nuanced and diversified cognitive processing of technical topics, which naturally generates complex and often contradictory affective evaluations. In contrast, the “mass-market sustainability” narrative emphasized by Brand_B cultivates a shared identity grounded in practical value and ecological responsibility. Users within this community show a greater tendency to engage via the peripheral route, relying on affective heuristics and practical outcomes such as range and cost for evaluation. This leads to the formation of concentrated and stable positive emotion, which is continuously reinforced through ongoing social identity processes.

### 5.2. Emotional Homophily and the Reinforcement Cycle of Network Structures

The cognitive-affective patterns initiated by brand narratives further crystallize into structurally distinct online interaction networks. Within the Brand_B community, high emotional homophily serves as the core organizing principle. Users who share similar positive emotions are more likely to form connections and, through ongoing interaction, continually reinforce their shared affect, resulting in high-density, high-modularity affective echo chambers. This network structure efficiently amplifies consistent narratives and rapidly solidifies a collective perception of reliability and high cost-effectiveness, though it may simultaneously suppress critical discourse. The Brand_T community presents a contrasting picture: the affective diversity stemming from deep cognitive processing yields a more fragmented and less modular network structure.

### 5.3. Behavioral Mechanisms Driving Tie Formation

The Exponential Random Graph Model findings elucidate the behavioral rules shaping the aforementioned network structures at a micro-mechanistic level.

The effect of perceived benefit on tie formation is consistent and strong in both communities. It transcends the traditional individual “risk-benefit” decision-making model, pointing toward a socialized benefit-seeking behavior. In the context of adopting novel technologies with high uncertainty, sharing and acquiring information about benefits (e.g., real-world range, cost savings, driving experience) is a crucial social strategy for uncertainty reduction. Consequently, users with high benefit perception naturally become attractive hubs in the network: they actively disseminate positive signals (significant sender effect) and are sought after for their informational utility (significant receiver effect). This creates a positive feedback loop: the exchange of benefit information reinforces the collective belief in the technology value, which in turn incentivizes further benefit-centered social interaction. The stronger effect in the Brand_B community likely aligns with its pragmatic brand positioning, where users are more focused on optimizing tangible ownership and usage experiences through information exchange.

The finding that users employing cognitive language (information contributors) exhibit high tendencies to both send and receive, yet avoid connecting with each other (negative homophily effect), offers a profound insight into the structure of the information ecosystem within online communities. It delineates a clear asymmetric division of labor: Cognitive Contributors versus Information Seekers. Contributors utilize analytical thinking to process information and serve the broader community, evidenced by a strong sender effect. Concurrently, they rely on gathering raw feedback and questions from a diverse base of general users, corresponding to a significant receiver effect. The relative lack of connections among contributors, however, may indicate their tendency to avoid redundant information exchange or the presence of subtle knowledge-status competition.

The ERGM results ultimately corroborate and mechanistically explain the network structural differences arising from brand narratives. In the Brand_B community, emotional homophily is the core organizing principle, highly consistent with a social identity based on shared values (pragmatism, eco-friendliness). In the Brand_T community, ties are driven more by social connection, with weak emotional homophily. Users may connect through debate, questioning, or challenge, explaining why shared sentiment is not a prerequisite. This fundamental difference in drivers indicates that the Brand_B community builds trust and cohesion through affective solidarity, whereas the Brand_T community sustains itself as a dynamic marketplace of ideas through ongoing public participation and cognitive expression.

### 5.4. Theoretical Contributions and Practical Implications

These findings have important theoretical implications. Traditional technology acceptance models (e.g., TAM, UTAUT) emphasize perceived usefulness and ease of use as cognitive determinants of adoption (Venkatesh et al., 2003). Our study complements these frameworks by demonstrating the role of affective and social dynamics in shaping user engagement before formal adoption takes place. Emotional homophily and sentiment clustering may function as early-stage behavioral signals that mediate collective perception of NEV technologies, which can accelerate or hinder diffusion.

The study bridges the gap between micro-level information processing and macro-level group phenomena by situating the Elaboration Likelihood Model and Social Identity Theory within the structural context of social networks. We demonstrate how technology perception emerges from individual cognitive-affective processes into a group-level, structured property. We find that while central route processing is associated with deep cognition, it may lead to affective heterogeneity and network fragmentation within communities; conversely, peripheral route processing, when coupled with a strong shared social identity, can efficiently drive emotional homophily and the formation of tight-knit communities. This deepens the understanding of the social nature of technology adoption, suggesting that successful brand communication depends not only on providing information or evoking emotion but also on whether its narrative can guide the formation of a social network structure conducive to the diffusion of consumer perceptions. This brand-mediated affective divergence offers a new angle for understanding consumer segmentation beyond demographics or psychographics.

From a policy and communication perspective, these insights highlight the need for emotionally informed engagement strategies. Automotive firms and public agencies should design technological narratives that not only convey functional attributes but also align emotionally with target user communities. For brands seeking to build highly cohesive and trusting communities, it is crucial to strengthen shared-value narratives that evoke emotional resonance and nurture positive sub-community ecosystems. In emotionally fragmented settings, efforts to encourage dialogue across sentiment groups, such as promoting inclusive storytelling or reducing algorithmic filtering, may help counteract polarization and foster more balanced evaluations.

### 5.5. Limitations

This study has several limitations that also point to directions for future research. First, the data are drawn from a single automotive forum and are cross-sectional in nature. Subsequent work could expand on this by adopting longitudinal designs, incorporating multilingual sentiment models, or combining behavioral trace data with survey-based psychological profiling. Such approaches would allow researchers to capture the dynamic evolution of sentiment and network structures, particularly around critical junctures such as product launches or safety incidents, thereby offering deeper insights into the volatility or resilience of consumer emotion in digital contexts.

Second, conducting cross-platform comparative studies and experimental designs could provide causal insights into the roles of emotional contagion, informational diversity, and behavioral nudges, while also helping to move beyond the constraints of lexicon-based sentiment analysis. Furthermore, while this study addresses differences in brand narratives, it does not quantitatively examine the role of platform affordances such as algorithmic recommendations, content moderation, and user anonymity. Future research that integrates these factors would help clarify how structural design influences emotional alignment and interaction behaviors.

Overall, this study contributes to the growing field of transportation psychology by linking emotional dynamics, cognitive framing, and peer interaction structures in NEV-related digital environments. It provides a methodological and theoretical foundation for understanding how online communities shape technology perception in the emerging era of sustainable mobility.

## Figures and Tables

**Table 1:** Summary Statistics of Brand_B and Brand_T Forum Interactions.

	Brand_B	Brand_T
Number of Forums	21	4
Total Users	2592	2079
Number of Posts	617	432
Number of Replies	5751	4835

**Table 2:** Variables and illustrative words for attributes.

Variables	Examples
Emotion	happy, cried, hate
Social connection	Mate, talk, his
Health perception	clinic, flu, pill
Risk perception	danger, accident, doubt
Benefit perception	efficient, eco-friendly, benefit
Cognition	think, know, because
Informal language	thx, umm, Imean

**Table 3:** Discussion Topics of Brand_B Forum Users (LDA Results).

	Topics	Keywords	Percentage
Topic 1	Experience and Evaluation	Feel, Tires, Sound System, Screen, Space	7.80%
Topic 2	Car Model Technology	Tang, Dolphin, Car Models, Characteristics, Performance	59.03%
Topic 3	Technical Performance and Safety	Body, Structure, Safety, Collision Avoidance, Insurance	3.95%
Topic 4	Technological Innovation	Battery, Range, Power Consumption, Motor, Chip	18.66%
Topic 5	Maintenance and Repair	Brake Pads, Calipers, Original Vehicle, Brake Discs, Replacement	6.12%
Topic 6	Auto parts and Modification	Accessories, Cameras, Wires, Customization, Modification	4.44%

**Table 4:** Discussion Topics of Brand_T Forum Users (LDA Results).

	Topics	Keywords	Percentage
Topic 1	Charging Facilities	Charging Pile, Electric Meter, Station, Installation, Facilities	7.75%
Topic 2	Battery Performance and Power Consumption Analysis	Battery, Energy Consumption, Charge, Motor, Transmission	4.32%
Topic 3	Electric Vehicle Performance and Range	Range, Electric, Mileage, Energy Consumption, Battery	8.83%
Topic 4	Vehicle Data Management and Systems	Data, System, Monitoring, Software, Technology	10.36%
Topic 5	Tires and Vehicle Maintenance	Tires, Maintenance, Hub, Repair, Maintenance	4.41%
Topic 6	Sound Insulation and Vehicle Improvement	Sound Insulation, Improvement, Price, Quietness, Design	5.41%
Topic 7	Interior Design and Functionality	Interior, Space, Configuration, Function, Interface	5.14%
Topic 8	Braking System and Safety	Brakes, Safety, Maintenance, Testing, Features	5.14%
Topic 9	Vehicle Accessories and Configurations	Vehicle-included Iools, Tools, Parts, Configuration, Accessories	6.13%
Topic 10	Air Conditioning System and Impact on Range	Air Conditioning, Range, Energy Consumption, System, Temperature Control	6.58%
Topic 11	After-Sales Service and Customer Experience	After-sales, Car Owner, Experience, Insurance, Service	10.54%
Topic 12	International Market and Cost Analysis	USA, Shanghai, Competition, Market, Cost	14.14%
Topic 13	Intelligent Connectivity System	Driving, Upgrade, Software, Autonomous, Cruise	11.26%

**Table 5:** ERGM Estimates of Behavioral Interaction Drivers in NEV Forums.

	Homophily		Receiver Effect		Sender Effect	
	Brand_B	Brand_T	Brand_B	Brand_T	Brand_B	Brand_T
edges	−5.55 *** (0.02)	−6.97 *** (0.02)	−7.11 *** (0.04)	−7.22 *** (0.05)	−7.22 *** (0.05)	−7.37 *** (0.05)
Emotion	2.30 ** (0.78)	−0.60 (0.59)	0.36 *** (0.05)	0.28 *** (0.06)	0.45 *** (0.04)	0.21 ** (0.07)
Social connection	−1.27 *** (0.11)	0.65 *** (0.09)	0.00 (0.00)	−0.02 *** (0.01)	0.00 (0.00)	0.02 *** (0.01)
Health perception	0.07 (0.04)	0.12 ** (0.04)	−0.08 *** (0.01)	−0.04 ** (0.01)	−0.05 ** (0.01)	−0.06 *** (0.01)
Risk perception	−1.78 *** (0.06)	−0.20 *** (0.05)	−0.00 (0.00)	−0.04 ** (0.01)	−0.00 (0.00)	−0.04 *** (0.01)
Benefit perception	1.72 *** (0.06)	0.27 *** (0.05)	0.02 *** (0.01)	0.04 *** (0.01)	0.00 (0.00)	0.03 *** (0.01)
Cognition	−0.93 * (0.38)	−1.04 (1.01)	0.01 *** (0.00)	0.02 *** (0.00)	0.01 *** (0.00)	0.02 *** (0.00)
Informal language	0.01 (0.20)	−0.67 * (0.33)	−0.02 *** (0.00)	−0.00 (0.00)	−0.01 *** (0.00)	−0.00 (0.00)
AIC	−1,766,455.35	−2,522,789.01	−2,562,624.86	−1,354,735.90	−2,039,268.68	−2,716,487.49
BIC	−1,766,355.58	−2,522,669.50	−2,562,502.09	−1,354,616.39	−2,039,148.91	−2,716,367.98
Log Likelihood	883,236.67	1,261,403.51	1,281,321.43	677,376.95	1,019,643.34	1,358,252.74

Note: The results are based on ERGM analysis. Significant levels are denoted as follows: *** *p* < 0.001; ** *p* < 0.01; * *p* < 0.05.

## Data Availability

The datasets presented in this article are not readily available because the data are part of an ongoing research project and involve substantial data collection costs. Requests to access the datasets should be directed to the luliangdong@hhu.edu.cn.
